# Five-year follow-up of the OptiTrain trial on concurrent resistance and high-intensity interval training during chemotherapy for patients with breast cancer

**DOI:** 10.1038/s41598-024-65436-z

**Published:** 2024-07-03

**Authors:** Poorna Anandavadivelan, Sara Mijwel, Maria Wiklander, Philippe Lee Meeuw Kjoe, Maryse Luijendijk, Jonas Bergh, Helene Rundqvist, Yvonne Wengstrom

**Affiliations:** 1 Department of Neurobiology, Care Sciences and Society, Division of Nursing, Karolinska Institute, Stockholm, Sweden; 2 Department of Physical Performance, The Norwegian School of Sport Sciences, Oslo, Norway; 3 Division of Psychosocial Research and Epidemiology, The Netherlands Cancer Institute-Antoni Van Leeuwenhoek, Amsterdam, The Netherlands; 4 Department of Oncology-Pathology, Karolinska Institutet, Stockholm, Sweden; 5 Breast Center, Karolinska University Hospital, Karolinska Comprehensive Cancer Center, Stockholm, Sweden; 6 Department of Laboratory Medicine, Karolinska Institutet, Stockholm, Sweden; 7 Unit of Clinical Physiology, Karolinska University Hospital, Stockholm, Sweden

**Keywords:** Breast cancer, Exercise, Fatigue, Adjuvant chemotherapy, Physical activity, Long-term effects, Cognitive assessment, Cancer, Breast cancer

## Abstract

The protocol predefined aim of this study is to assess sustained effects of the OptiTrain trial on several health outcomes, 5 years after the baseline assessment. The OptiTrain study was a prospective, randomised controlled trial with 240 patients with breast cancer undergoing adjuvant chemotherapy that compared the effects of 16 weeks of two exercise programs, RT-HIIT and AT-HIIT, with usual care (UC). After a 5-year follow-up, eligible participants were evaluated for the primary outcome of cancer-related fatigue (CRF) and secondary outcomes including quality of life, symptoms, muscle strength, cardiorespiratory fitness, body mass, physical activity, and sedentary behavior. Statistical analysis was conducted using linear mixed models adjusted for baseline values. Tumour profile and menopausal status were additionally adjusted for CRF. Mean differences (MD), 95% confidence intervals (CIs), and standardized effect sizes (ES) were reported. At the 5-year follow-up, there were no statistically significant differences in total CRF between the intervention groups and the UC group. RT-HIIT reported significantly reduced pain sensitivity at the gluteus MD = 79.00 (95% CI 10.17, 147.83, ES = 0.55) compared to UC. Clinically meaningful differences for an increase in cognitive CRF and cardiorespiratory fitness were observed for the AT-HIIT versus UC group, and for lower limb strength for the RT-HIIT versus UC group, albeit without statistical significance. Engaging in targeted exercise during adjuvant chemotherapy for breast cancer provides short-term benefits in reducing fatigue and maintaining physical function. However, our 5-year follow-up indicates that these effects are limited in the long term. This underscores the need to support breast cancer survivors maintain their PA levels throughout their survivorship journey.

## Introduction

Benefits of physical activity (PA) is well-established in improving the well-being of women with breast cancer, especially in alleviating treatment-related side effects^1–3^. While short-term benefits of PA in patients with cancer are well-documented, knowledge regarding its long-term effects still remains limited^4,5^. Specifically, only a few randomized controlled trials (RCT) within exercise oncology have follow-up periods extending beyond two years following the intervention^6–8^. Data suggest lasting benefits into survivorship, particularly an increase in physical activity levels (PAL) from the initial intervention. While both aerobic and resistance exercises have individually shown positive health effects for women with breast cancer^9,10^, cancer-specific exercise guidelines recommend a structured program that incorporates both resistance and aerobic exercises^11^. Moreover, despite the potential promise of resistance training and combined resistance and aerobic approaches in improving physical fitness outcomes for breast cancer survivors^9,12^, no study has yet examined the long-term effects of two distinct exercise regimens within a trial. Understanding the lasting effects of exercise programs that adhere to the FIIT principles (frequency, intensity, type, and time) is crucial for developing sustainable, meaningful fitness routines for individuals after a breast cancer diagnosis and treatment. Taking these aspects into consideration, the OptiTrain RCT compared two distinct 16-week supervised exercise programs (resistance exercise and high-intensity interval training (HIIT) and moderate-intensity aerobic exercise and HIIT) on patient-reported and physiological outcomes in women with breast cancer undergoing chemotherapy. Our previous findings from the OptiTrain RCT^13^ revealed short- and long-term positive effects, comprising data from immediately after the intervention and up to two years following the intervention on patient-reported and physiological outcomes^14–17^. The present study is the 5-year follow-up of the 16-week OptiTrain RCT. We hypothesized that the effects of the OptiTrain intervention will be sustained at five years following the completion of the intervention. Hence, the protocol predefined aim was to assess the sustained effects of differences in the primary outcome of cancer-related fatigue (CRF) and secondary outcomes of health-related quality of life (HRQoL), symptoms, muscle strength, cardiorespiratory fitness, body mass, PAL and return to work between the two OptiTrain exercise groups and the usual care (UC) group, five years after the baseline assessment. Additionally, we examined whether groups differed in their cognitive functioning five years post-baseline.

## Methods

### Study design and setting

The OptiTrain RCT design and setting have been extensively detailed in the original protocol^13^ and prior findings^14–17^. It was a prospective, in-clinic RCT with three arms, comparing 16 weeks of concurrent resistance and high-intensity interval training (RT-HIIT) or concurrent moderate-intensity aerobic exercise and high-intensity interval training (AT-HIIT) to a control group receiving usual care (UC). Participants were enrolled from Stockholm oncology clinics between March 2013 and July 2016. Eligible women (ages 18–70, early breast cancer stages I-IIIa) undergoing adjuvant chemotherapy were randomly assigned to groups. The Clinical Studies Unit at Karolinska University Hospital randomly assigned participants to one of three groups: RT-HIIT, AT-HIIT, or UC, using a computer program for random assignment at a 1:1:1 ratio. This allocation process was blinded to the research team. Prior to the initial assessment, participants, exercise supervisors, and outcome assessors were informed of participant’s group allocation and were not blinded to it. This study reports data from the 5-year follow-up of the 16-week OptiTrain RCT and is part of the original protocol^13^.

### Ethical considerations

The study procedures were performed in accordance with the ethical standards of the institutional and national research committee (Regional Ethical Review Board in Stockholm, Sweden) that approved the study (registration numbers: 2012/1347–31/1, 2012/1347- 31/2, 2013/7632–32, 2014/408–32, 2016/57–32, 2018/446/32) and in accordance with the 1964 Helsinki declaration and its later amendments. The OptiTrain trial has been registered at ClinicalTrials.gov (NCT02522260 first registered 13/08/2015, Optimal Training for Women with Breast Cancer (OptiTrain), http://www.clinicaltrials.gov). Informed consent was obtained from all participants who were included in the study. After five years from the baseline assessment, eligible participants were contacted and invited to attend an in-clinic assessment session and to complete online questionnaires and cognitive tests.

### Outcome measures

An overview of the outcome measures used at the various assessment time points of the OptiTrain RCT is presented in Table 1. The primary outcome was cancer-related fatigue, and the secondary outcomes were symptoms and symptom burden, health-related quality of life, cardiorespiratory fitness, lower and upper body muscle strength, pressure pain threshold (PPT), objective measures of sedentary behaviour and physical activity, body mass, sick leave, heart failure diagnosis and cognitive functioning. Most outcomes were assessed at all time points: baseline (1 week before the second chemotherapy session), post-intervention (16 weeks after the baseline), 1-year post-baseline, 2 years post-baseline, and 5 years post-baseline. The exceptions to this were objectively measured PA which was assessed at baseline, 2 years, and 5 years only; the assessment of pressure pain threshold (PPT) that occurred at baseline, 16 weeks, 2 years, and 5 years only; and heart failure diagnoses and objectively measured cognitive functioning which were measured only at 5 years.

**Table 1:** Outcome measures used in OptiTrain RCT at baseline and at 16 weeks, 1 year, 2 year and 5 years follow-up post-baseline.

Outcome	Tool used	Baseline	16 weeks post baseline	1 year post baseline	2 years post baseline	5 years post baseline
Cancer-related fatigue*	Swedish version of Revised Piper fatigue Scale (PFS) Swedish version^44^	×	×	×	×	×
Symptoms and symptom burden**	Swedish version of Memorial Symptom Assessment Scale (MSAS)^45,46^	×	×	×	×	×
Health-related quality of life**	Swedish version of European Organisation for Research and Cancer Treatment Quality of Life Questionnaire (EORTC-QLQ-C30) Version 3.0^47^	×	×	×	×	×
Cardiorespiratory fitness**	Åstrand-Rhyming submaximal cycle test (Monark 928E, Monark Exercise AB, Vansbro, Sweden)^48,49^	×	×	×	×	×
Lower body muscle strength**	Isometric mid-thigh pull (Baseline leg dynamometer, Fabrication Enterprises Inc., White Plains, NY, USA)^50^	×	×	×	×	×
Upper body muscle strength**	Hand grip tests (JAMAR, SAEHAN corporation, Changwon, S. Korea)	×	×	×	×	×
Pressure Pain Threshold (PPT)**	Electronic algometer (Somedic Sales AB, Hörby, Sweden)^51^	×	×	–	×	×
Objective measures of Sedentary behaviour and physical activity**	Accelerometer (model GT3X ActiGraph® Corp, Pensacola, Florida, USA)^22^	×	–	–	×	×
Body mass**	Calibrated electric scales	×	×	×	×	×
Sick leave**	Single item study specific questionnaire	×	×	×	×	×
Heart failure diagnosis**	Swedish Heart Failure Registry^18^	–	–	–	–	×
Cognitive functioning**	Amsterdam Cognition Scan (ACS)^19,20^	–	–	–	–	×

*Primary outcome; **Secondary outcomes.

More comprehensive information regarding the methods for the outcome measures has been presented in previous publications^14–17^. Participants were asked to complete a single-item questionnaire on their sick leave status, with five options: 0% (no sick leave), 25%, 50%, 75%, or 100% (full-time sick leave). Information on heart failure (HF) diagnosis was obtained from the Swedish Heart Failure Registry^18^. Cognitive functioning was measured using the Amsterdam Cognition Scan (ACS), an online validated neuropsychological test battery^19,20^, which participants completed on their own computer at home. An overview of ACS tests, outcome measures and corresponding cognitive domains are found in Table 2.

**Table 2:** Cognitive tests of the Amsterdam cognition scan.

Cognitive domain	ACS test	Main outcome measure
Learning and memory	Wordlist learning	Total number of correct words (learning: trial 1 to 5)
	Wordlist recall	
	Wordlist recognition	
Attention and working memory	Box tapping	Total number of correctly repeated sequences
	Digit sequences I	
	Digit sequences II	
Processing speed	Reaction speed	Average reaction time (ms)
	Connect the dots I	Completion time in seconds
Executive functioning	Connect the dots II	Completion time in seconds
	Place the beads	Total number of extra moves
Motor functioning	Fill the grid	Completion time in seconds

### Supervised exercise program

The 16-week OptiTrain exercise intervention has been described in previous publications^14–17^. In summary, both groups trained twice a week on non-consecutive weekdays for 16 weeks. Each session lasted approximately 60 min and was carried out at the exercise clinic at Karolinska University Hospital. An exercise physiologist or oncology nurse supervised all sessions to ensure safety, correct technique, and adherence to the exercise protocols. Each exercise session started with a 5-min warm-up on a cycle ergometer or treadmill at a Borg scale rate of perceived exertion (RPE) of 10–12^21^ and concluded with a 10-min cool-down involving dynamic muscle stretching.

The RT-HIIT group performed eight resistance exercises targeting major muscle groups using using weight stack training equipment, participants’ body mass, free weight dumbbells or barbells. Exercises included leg presses, bicep curls, squat jumps, tricep extensions, lunges, bench presses, sit-ups or Russian-weighted abdominal twists, shoulder presses, and prone-lying back extensions. Participants started with 2 sets of 8–12 repetitions at 70–80% of their estimated one-repetition maximum (1-RM). The RT-HIIT sessions concluded with three 3-min bouts of HIIT at a perceived exertion rating (RPE) of 16–18, with one minute of recovery between bouts on a cycle ergometer.

The AT-HIIT group began each session with 20 min of moderate-intensity (RPE 13–15) continuous aerobic exercise, followed by the same HIIT regimen as the RT-HIIT group.

Attendance was calculated as the percentage of completed sessions, and adherence was determined based on participants successfully completing 90% of the planned exercise sessions, accounting for intensity and duration, within the intervention groups.

### Follow‑up period

After the 16-week exercise program, participants received written prescriptions for both aerobic and resistance exercises. One-on-one counseling sessions with a health educator were offered, along with reduced-rate gym memberships. Over the 2-year follow-up (2014–2017), participants attended seven educational sessions covering topics like healthy lifestyles, fitness, and exercise programs to promote motivation and continued engagement.

### Usual care

The control group (UC) received care as usual for breast cancer and written information on physical activity at the start of the intervention period, including exercise recommendations for cancer patients based on American College of Sports Medicine guidelines^11^, but no supervised exercise training or specific exercise prescriptions.

### Sample size calculation

The initial power calculation used total fatigue (Piper Fatigue Scale) as the primary outcome after the 16-week intervention. With an effect size of 0.53 and a desired power of 0.8, 65 participants per group were needed. To account for a 20% anticipated dropout rate, 80 participants were recruited for each group.

### Statistical analysis

Baseline demographics were summarized for all participants, including those who remained and those who dropped out. Summary statistics including means, standard deviations, frequencies, and percentages were utilized to conduct a descriptive analysis. Between group differences were calculated using linear mixed model (LMM) analysis using outcome measurements collected at 16 weeks (post-intervention), 1 year, 2 years, and 5 years after the baseline assessment adjusting for baseline values for all variables. For the primary outcome of CRF, tumour profile and menopausal status were also additionally adjusted for in the analysis based on knowledge of known prognostic factors. At the 5-year follow-up, completers were defined as those participants who had at least one non-missing response to the questionnaires at the 5-year assessment. For cardiorespiratory fitness outcomes, measured as predicted peak oxygen uptake (VO2_peak_) estimated VO2_peak_ (L⋅min^−1^) and estimated VO2_peak_ (mL⋅kg^−1^⋅min^−1^)], measurements above physiologically acceptable cut-off limits of 65 mL⋅kg^-1^⋅min^−1^) were removed from the linear mixed models (N = 6; 5-year follow-up = 3; 2-year follow-up = 2; 1 year-follow-up = 1) considering also if the changes from the previous timepoints are significantly large to be acceptable. The outcome objectively assessed PA and sedentary behavior were analysed using validated wear-time specifications and cut-offs for adults^22^. Between-group differences in cognitive functioning 5 years after baseline were assessed using one-way ANOVA, after excluding outliers for ACS outcomes. For time-based outcomes (Connect the dots I and II, Reaction speed, Fill the grid), the median absolute deviation (MAD)^23^ method was used for outlier detection. The MAD was applied times 3.5 per age group (< 50 years, 50–59 years, ≥ 60 years). For count outcomes (Wordlist learning, Box tapping, Digit sequences I and II), 0 scores were excluded as these most likely reflect technical or human errors. Scores on Wordlist recall and recognition were excluded if the retention interval was longer than 45 min.

Mean differences and 95% confidence intervals (CIs) were reported, along with standardized effect sizes (ES) that were calculated and interpreted as described previously^24,25^. A significance level of *p* < 0.05 was used for all statistical analyses. All statistical analysis was performed using the statistical software IBM SPSS Statistics Version: 28.0.1.1 (14).

## Results

**Figure 1:**
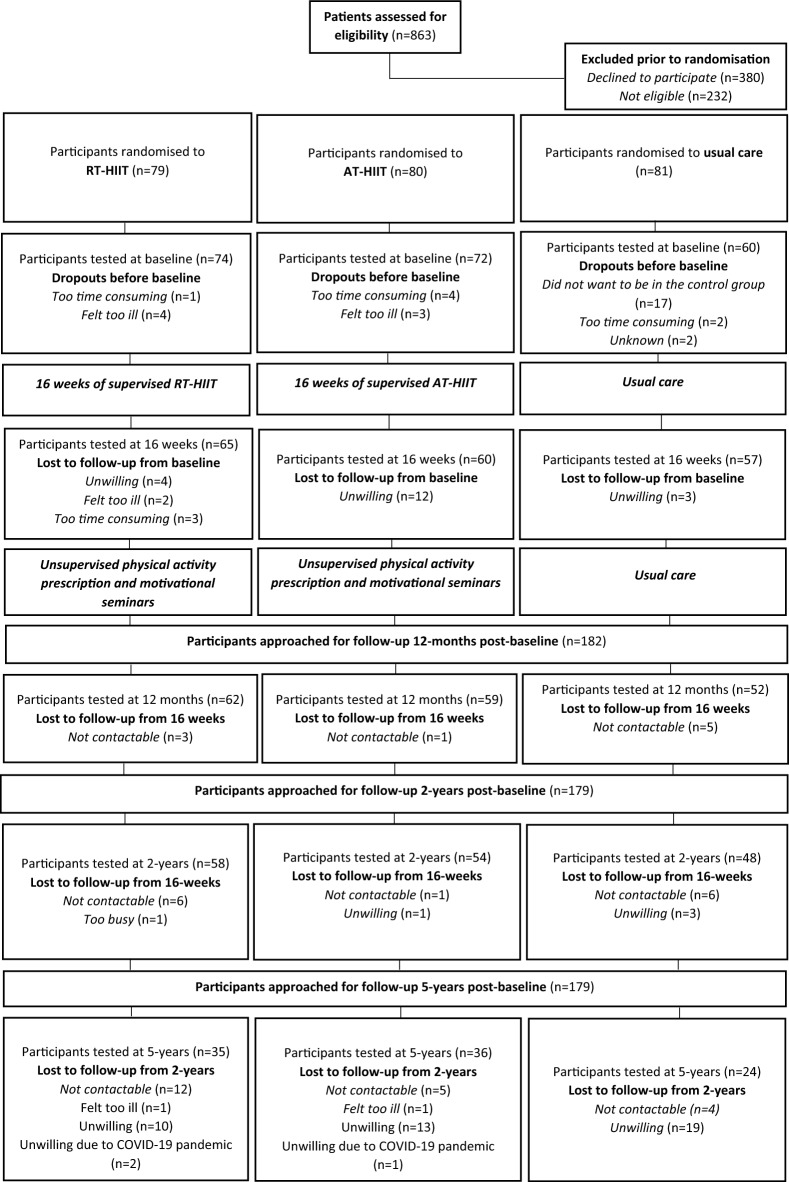
CONSORT flowchart illustrating the participant progression throughout the OptiTrain study.

The flow diagram (Fig. 1) illustrates the progress of all participants throughout the study. At the 5-year post-baseline timepoint, a total of 179 survivors were eligible and invited to take part in the assessments. Among those, 95 participants (*N*_*RT-HIIT*_ = 35; *N*_*AT-HIIT*_ = 36; *N*_*UC*_ = 24); 40% of the initially randomized individuals and 46% of those who completed baseline testing) answered the questionnaires, while 97 participants (*N*_*RT-HIIT*_ = 33; *N*_*AT-HIIT*_ = 34; *N*_*UC*_ = 30; 40% of the initially randomized individuals and 47% of those who completed baseline testing) underwent at least one of the in-clinic physiological assessments. Nearly, 67 participants completed cognitive testing (*N*_*RT-HIIT*_ = 24; *N*_*AT-HIIT*_ = 27; *N*_*UC*_ = 16). Among those who were eligible and invited, despite not participating in the assessments at the 2-year follow-up, 10 additional participants returned for the 5-year assessments. About, 68 participants who answered the questionnaires at the 2-year follow-up did not answer the 5-year questionnaires and were therefore considered dropouts.

Socio-demographic and clinical characteristics of the participants at baseline of the OptiTrain RCT and those who completed the 5-year follow-up and included in the present study are provided in Table 3. No statistical test was performed to compare these characteristics as they may necessarily not indicate the presence or absence of confounding factors or biases^26^. Similarly, characteristics of those who answered the 2-year follow-up but not the 5-year questionnaires is presented in Table 4 (no statistical test were performed to compare these characteristics). At the 5-year follow-up, a higher proportion in the RT-HIIT group had higher MVPA (90.8 ± 34.9), higher education (77.1%) and less frequent triple negative breast cancer (5.7%); the AT-HIIT group were more often post-menopausal (77.8%); a higher proportion in the UC group were married/partnered (79.2%) (Table 3). Besides this, a higher percentage among all three groups at the 5-year follow- up received taxane-based therapy compared to all participants and the dropouts (Tables 3, 4).

**Table 3:** Baseline characteristics of all OptiTrain participants and completers 5-years post-baseline.

	All participants tested at baseline n = 206			Completers 5 years post-baseline n = 95		
	RT-HIIT n = 74	AT-HIIT n = 72	UC n = 60	RT-HIIT n = 35	AT-HIIT n = 36	UC n = 24
Age (years)	52.7 ± 10.3	54.4 ± 10.3	52.6 ± 10.2	53.5 ± 10.4	56.1 ± 8.3	52.8 ± 9.4
Body mass (kg)	68.7 ± 11.3	67.7 ± 13.0	69.1 ± 11.0	68.1 ± 9.5	67.2 ± 11.7	69.6 ± 9.6
BMI (kg/m2)	25.1 ± 4.3	24.8 ± 4.4	24.6 ± 4.8	24.7 ± 3.7	24.7 ± 3.9	24.7 ± 4.3
MVPA (min/week)	79.8 ± 31.7	70.4 ± 28.9	70.0 ± 36.4	90.8 ± 34.9	76.1 ± 26.0	71.6 ± 38.6
SED (min/day)	523.3 ± 112.7	543.6 ± 109.0	552.8 ± 101.9	535.3 ± 103.0	562.4 ± 107.7	576.6 ± 87.9
Married or partnered	60.6	59.7	69.5	60.0	58.3	79.2
University completed	67.6	64.7	66.0	77.1	66.7	54.2
Current smoker	4.3	5.9	5.2	2.9	2.8	4.2
Postmenopausal	51.4	63.9	61.7	54.3	77.8	54.2
Tumour profile						
Triple negative	14.9	11.0	16.7	5.7	16.7	16.7
HER2 + , ER + / −	21.6	30.2	20.0	25.7	33.4	20.9
HER2 − , ER +	62.2	58.9	61.6	65.7	49.9	58.2
HER2 − , ER −	1.4	0.0	1.7	2.9	0.0	4.2
Chemotherapy received						
Taxane based therapy	40.6	37.0	41.7	65.7	66.7	62.5
Anthracycline based therapy	59.4	63.0	58.3	34.3	33.3	37.5

Continuous variables are presented as mean ± SD, whereas dichotomous or categorical variables are presented as %, RT-HIIT resistance training and high-intensity interval exercise group, AT-HIIT moderate-intensity and high-intensity interval training group, UC usual care, BMI body mass index, MVPA objectively measured moderate- to vigorous-intensity physical activity, SED objectively measured sedentary behaviour, HER + /- human epidermal growth factor receptor 2 positive ( +) or negative (-), ER + /- estrogen receptor positive ( +) or negative (-), SD standard deviation.

**Table 4:** Baseline characteristics of dropouts after 2 years post-baseline.

	Completers at 2 years post baseline but not at 5 years post-baseline n = 68		
	RT-HIIT n = 25	AT-HIIT n = 20	UC n = 23
Age (years)	52.4 ± 9.7	51.8 ± 10.1	53.8 ± 10.6
Body mass (kg)	68.3 ± 12.8	65.9 ± 16.7	68.5 ± 12.6
BMI (kg/m2)	25.0 ± 4.5	23.8 ± 5.6	25.2 ± 4.6
MVPA (min/week)	74.0 ± 27.3	69.4 ± 34.7	64.6 ± 28.6
SED (min/day)	534.9 ± 122.5	523.7 ± 108.6	552.0 ± 77.9
Married or partnered	64.0	65.0	60.9
University completed	52.0	75.0	69.6
Current smoker	0.0	10.0	4.3
Postmenopausal	52.0	40.0	65.2
Tumour profile			
Triple negative	16.0	5.0	21.7
HER2 + , ER + / −	24.0	25.0	21.7
HER2 − , ER +	56.0	70.0	56.5
HER2 − , ER −	4.0	0.0	0.0
Chemotherapy received			
Taxane based therapy	52.0	50.0	52.2
Anthracycline based therapy	48.0	50.0	47.8

Continuous variables are presented as mean ± SD, whereas dichotomous or categorical variables are presented as %, RT-HIIT resistance training and high-intensity interval exercise group, AT-HIIT moderate-intensity and high-intensity interval training group, UC usual care, BMI body mass index, MVPA objectively measured moderate- to vigorous-intensity physical activity, SED objectively measured sedentary behaviour, SD standard deviation.

In the Swedish Heart Failure Registry, HF diagnoses were registered for four study participants. Moreover, two additional cases of HF were diagnosed during the follow-up assessments of the RCT.

The attendance rates for the supervised exercise intervention were 68% for the RT-HIIT group and 63% for the AT-HIIT group. Regarding adherence to the exercise prescription in the supervised exercise intervention, it was 83% for the RT-HIIT group and 75% for the AT-HIIT group. On average, the attendance for the seven motivational seminars was twenty percent, with a range of 11% to 27%.

### Primary outcome: cancer-related fatigue

The between-group differences of the mean scores (mean differences = MD) for the main outcome of CRF as assessed by using PFS is presented in Table 5. At 5 years, no group showed an average total fatigue severity cut-score above 3 (mild), and no statistically significant nor clinically meaningful differences were observed for total cancer-related fatigue between either of the intervention groups and the UC group; RT-HIIT vs UC: MD =

**Table 5:** Cancer-related fatigue 5 years post-baseline.

		Baseline Mean ± SD	16 weeks Mean ± SD	2 years Mean ± SD	5 years Mean ± SD		Baseline to 5 years Between-group differences Mean difference (95% CI)	ES
Piper Fatigue Scale								
Total CRF	RT-HIIT	3.14 ± 3.18	3.12 ± 3.03	2.92 ± 2.76	2.55 ± 3.12	RT-HIIT versus UC	− 0.02 (− 1.50, 1.46)	0.10
	AT-HIIT	2.10 ± 2.63	3.18 ± 2.77	2.34 ± 2.63	2.37 ± 2.54	AT-HIIT versus UC	0.14 (− 1.34, 1.62)	0.43
	UC	2.42 ± 2.90	3.98 ± 3.05	2.37 ± 2.70	1.51 ± 2.40			
Behaviour/daily life CRF	RT-HIIT	3.14 ± 3.38	3.01 ± 3.31	2.61 ± 2.89	2.54 ± 3.36	RT-HIIT versus UC	− 0.45 (− 1.96, 1.06)	0.02
	AT-HIIT	1.87 ± 2.57	2.98 ± 2.89	2.17 ± 2.70	2.01 ± 2.45	AT-HIIT versus UC	− 0.24 (− 1.75, 1.26)	0.30
	UC	2.13 ± 2.85	4.02 ± 3.29	2.75 ± 2.77	1.47 ± 2.48			
Emotional/affective CRF	RT-HIIT	3.33 ± 3.43	3.45 ± 3.33	3.30 ± 3.11	2.75 ± 3.33	RT-HIIT versus UC	0.13 (− 1.49, 1.75)	0.14
	AT-HIIT	2.37 ± 2.97	3.74 ± 3.23	2.51 ± 2.88	2.57 ± 2.81	AT-HIIT versus UC	0.28 (− 1.34, 1.90)	0.41
	UC	2.60 ± 3.15	4.24 ± 3.22	3.74 ± 2.87	1.55 ± 2.53			
Sensory/physical CRF	RT-HIIT	3.31 ± 3.25	3.27 ± 3.19	3.13 ± 2.89	2.63 ± 3.22	RT-HIIT versus UC	0.15 (− 1.41, 1.71)	0.13
	AT-HIIT	2.27 ± 2.87	3.53 ± 3.15	2.50 ± 2.86	2.61 ± 2.82	AT-HIIT versus UC	0.27 (− 1.29, 1.83)	0.48
	UC	2.75 ± 3.21	4.29 ± 3.31	3.65 ± 2.96	1.64 ± 2.65			
Cognitive CRF	RT-HIIT	2.85 ± 2.97	2.82 ± 2.84	2.73 ± 2.65	2.30 ± 2.84	RT-HIIT versus UC	0.08 (− 1.42, 1.58)	0.26
	AT-HIIT	1.88 ± 2.51	2.61 ± 2.39	2.24 ± 2.51	2.37 ± 2.58	AT-HIIT versus UC	0.38 (− 1.13, 1.88)	0.68
	UC	2.73 ± 2.79	3.47 ± 2.87	3.43 ± 2.82	1.42 ± 2.26			

SD standard deviation, CI confidence intervals, ES effect size, CRF cancer-related fatigue, RT-HIIT resistance training and high-intensity interval exercise group, AT-HIIT moderate-intensity and.

high-intensity interval training group, UC usual care, ES effect size, fatigue severity cut-scores: 0 = none, 1–3 = mild, 4–6 = moderate, 7–10 = severe.

*p < 0.0.

− 0.02 (95% CI − 1.50, 1.46, ES = 0.10) and AT-HIIT versus UC: MD = 0.14 (95% CI − 1.34, 1.62, ES = 0.43). Similarly, no statistically significant differences were found for the four sub-scales of the PFS between either of the exercise groups and UC (Table 5). However, clinically meaningful differences for an increase in cognitive CRF MD = 0.38 (95% CI − 1.13, 1.88, **ES = 0.68**) were noted for AT-HIIT compared to UC.

### Secondary outcomes

#### Symptoms and health-related quality of life

At 5 years, neither of the exercise groups showed statistically significant differences for total symptoms assessed by MSAS compared to UC; RT-HIIT versus UC: MD = − 0.05 (95% CI − 0.22, 0.12, ES = − 0.04); AT-HIIT vs UC: MD = − 0.10 (95% CI − 0.27, 0.07, ES = 0.07). Similarly, no statistically nor clinically significant differences were seen for either of the MSAS subscales (symptom burden, physical symptoms, and psychological symptoms (Table 6). No statistically significant differences were found between the groups for global QoL; RT-HIIT versus UC: MD =

**Table 6:** Symptoms and symptom burden 5 years post-baseline.

Memorial symptom assessment scale (MSAS)		Baseline Mean ± SD	16 weeks Mean ± SD	2 years Mean ± SD	5 years Mean ± SD		Baseline to 5 years Between-group differences Mean difference (95% CI)	ES
Total Symptoms	RT-HIIT	0.74 ± 0.53	0.74 ± 0.50	0.51 ± 0.39	0.45 ± 0.41	RT-HIIT versus UC	− 0.05 (− 0.22, 0.12)	− 0.04
	AT-HIIT	0.65 ± 0.41	0.76 ± 0.51	0.39 ± 0.31	0.41 ± 0.32	AT-HIIT versus UC	− 0.10 (− 0.27, 0.07)	0.07
	UC	0.59 ± 0.50	0.85 ± 0.60	0.40 ± 0.33	0.32 ± 0.36			
Symptom burden	RT-HIIT	0.91 ± 0.72	0.77 ± 0.62	0.62 ± 0.57	0.59 ± 0.66	RT-HIIT versus UC	0.05 (− 0.20, 0.31)	0.06
	AT-HIIT	0.75 ± 0.58	0.66 ± 0.59	0.44 ± 0.50	0.45 ± 0.44	AT-HIIT versus UC	− 0.12 (− 0.37, 0.14)	0.09
	UC	0.71 ± 0.71	0.89 ± 0.67	0.52 ± 0.53	0.35 ± 0.51			
Physical symptoms	RT-HIIT	0.74 ± 0.59	0.68 ± 0.57	0.35 ± 0.37	0.31 ± 0.38	RT-HIIT versus UC	− 0.08 (− 0.25, 0.10)	− 0.24
	AT-HIIT	0.67 ± 0.49	0.75 ± 0.59	0.32 ± 0.33	0.30 ± 0.30	AT-HIIT versus UC	− 0.12 (− 0.30, 0.06)	− 0.15
	UC	0.52 ± 0.56	0.77 ± 0.62	0.30 ± 0.35	0.23 ± 0.34			
Psychological symptoms	RT-HIIT	0.97 ± 0.76	1.02 ± 0.77	0.83 ± 0.74	0.88 ± 0.93	RT-HIIT versus UC	0.22 (− 0.13, 0.57)	0.39
	AT-HIIT	0.81 ± 0.64	0.88 ± 0.73	0.58 ± 0.56	0.63 ± 0.62	AT-HIIT versus UC	− 0.03 (− 0.38, 0.32)	0.30
	UC	0.81 ± 0.77	1.11 ± 0.83	0.58 ± 0.60	0.42 ± 0.54			

SD standard deviation, CI confidence interval, ES effect size, RT-HIIT resistance training and high-intensity interval exercise group, AT-HIIT moderate-intensity and high-intensity interval training group,

UC usual care, ES effect size.

*p < 0.05.

− 2.50 (95% CI − 12.46, 7.47, ES = − 0.05); AT-HIIT versus UC: MD = 1.05 (95% CI − 8.96, 11.05, ES = − 0.07). Likewise, no significant differences between the two exercise groups and UC were noted for the QoL subscales from EORTC QLQ-C30 (Table 7).

**Table 7:** Health-related quality of life 5 years post-baseline.

EORTC-QLQ-C30		Baseline Mean ± SD	16 weeks Mean ± SD	2 years Mean ± SD	5 years Mean ± SD		Baseline to 5 years Between-group differences Mean difference (95% CI)	ES
Global/quality of life	RT-HIIT	63.60 ± 24.81	63.85 ± 19.88	71.01 ± 23.80	73.71 ± 21.30	RT-HIIT versus UC	− 2.50 (− 12.46, 7.47)	− 0.05
	AT-HIIT	66.67 ± 20.90	63.75 ± 20.29	75.13 ± 18.89	76.34 ± 15.66	AT-HIIT versus UC	1.05 (− 8.96, 11.05)	− 0.07
	UC	67.96 ± 21.89	59.52 ± 19.62	75.35 ± 18.87	79.17 ± 17.11			
Physical functioning	RT-HIIT	89.52 ± 14.62	85.88 ± 16.31	91.48 ± 19.01	93.11 ± 14.55	RT-HIIT versus UC	0.64 (− 6.72, 8.01)	− 0.10
	AT-HIIT	89.98 ± 11.41	85.86 ± 15.37	92.45 ± 11.12	89.89 ± 14.25	AT-HIIT versus UC	− 1.54 (− 8.94, 5.85)	− 0.37
	UC	87.55 ± 16.80	76.91 ± 20.22	87.86 ± 17.56	92.75 ± 11.70			
Emotional functioning	RT-HIIT	67.61 ± 25.71	72.30 ± 22.92	72.30 ± 22.92	76.00 ± 23.92	RT-HIIT versus UC	1.01 (− 9.34, 11.36)	0.04
	AT-HIIT	74.42 ± 18.94	79.86 ± 16.19	79.86 ± 16.19	79.29 ± 17.73	AT-HIIT versus UC	0.77 (− 9.59, 11.12)	− 0.12
	UC	74.48 ± 23.96	69.35 ± 26.26	69.30 ± 26.03	81.88 ± 13.90			
Role functioning	RT-HIIT	59.55 ± 34.62	70.83 ± 28.27	87.93 ± 23.10	88.51 ± 22.81	RT-HIIT versus UC	− 3.38 (− 16.40, 9.64)	0.11
	AT-HIIT	67.61 ± 30.55	71.39 ± 26.59	90.74 ± 16.71	86.66 ± 23.79	AT-HIIT versus UC	− 4.44 (− 17.46, 8.58)	− 0.21
	UC	69.13 ± 28.47	54.46 ± 34.16	87.50 ± 22.13	94.50 ± 12.56			
Cognitive functioning	RT-HIIT	77.08 ± 25.88	78.19 ± 20.82	76.14 ± 26.88	81.40 ± 18.38	RT-HIIT versus UC	− 2.43 (− 11.43, 6.57)	− 0.24
	AT-HIIT	81.39 ± 20.60	79.72 ± 19.91	82.39 ± 15.98	81.83 ± 19.04	AT-HIIT versus UC	− 3.90 (− 12.95, 5.15)	− 0.43
	UC	77.59 ± 25.59	69.94 ± 27.42	81.90 ± 19.98	88.08 ± 11.49			
Social functioning	RT-HIIT	65.01 ± 29.71	61.76 ± 48.79	80.72 ± 26.65	88.06 ± 22.36	RT-HIIT versus UC	2.48 (− 9.78, 14.74)	0.11
	AT-HIIT	72.91 ± 24.26	72.78 ± 24.92	87.02 ± 19.06	85.20 ± 22.03	AT-HIIT versus UC	− 1.57 (− 13.87, 10.72)	− 0.28
	UC	71.22 ± 30.20	62.20 ± 28.34	83.68 ± 25.84	91.00 ± 19.55			
Fatigue	RT-HIIT	39.51 ± 29.74	37.58 ± 24.51	24.10 ± 23.17	23.11 ± 25.84	RT-HIIT versus UC	1.39 (− 8.85, 11.64)	0.04
	AT-HIIT	35.54 ± 23.28	38.52 ± 24.84	21.76 ± 20.11	22.37 ± 20.40	AT-HIIT versus UC	1.98 (− 8.31, 12.28)	0.17
	UC	36.03 ± 27.19	48.81 ± 25.58	24.27 ± 20.21	18.58 ± 16.04			
Nausea and vomiting	RT-HIIT	13.12 ± 16.43	5.15 ± 10.49	5.78 ± 11.94	2.40 ± 7.18	RT-HIIT versus UC	− 0.38 (− 3.85, 3.10)	− 0.15
	AT-HIIT	12.99 ± 18.15	5.83 ± 12.21	2.48 ± 9.95	1.43 ± 6.20	AT-HIIT versus UC	− 1.24 (− 4.74, 2.26)	− 0.19
	UC	8.28 ± 16.94	8.04 ± 21.08	4.52 ± 16.04	0.00 ± 0.00			
Pain	RT-HIIT	21.62 ± 24.82	21.32 ± 25.08	15.56 ± 19.23	12.40 ± 19.47	RT-HIIT versus UC	− 2.26 (− 14.83, 10.32)	− 0.23
	AT-HIIT	15.48 ± 22.40	16.95 ± 20.47	15.14 ± 21.04	20.06 ± 24.87	AT-HIIT versus UC	3.49 (− 9.10, 16.09)	0.33
	UC	17.32 ± 26.24	27.38 ± 29.89	21.21 ± 26.77	13.88 ± 21.14			
Dyspnoea	RT-HIIT	24.78 ± 27.29	35.29 ± 29.86	20.65 ± 22.35	26.35 ± 22.93	RT-HIIT versus UC	3.82 (− 12.05, 19.68)	0.13
	AT-HIIT	22.25 ± 22.46	37.22 ± 28.19	22.17 ± 25.05	27.38 ± 27.85	AT-HIIT versus UC	4.35 (− 11.56, 20.26)	0.29
	UC	28.07 ± 25.54	44.05 ± 29.89	22.16 ± 23.13	26.29 ± 24.09			
Insomnia	RT-HIIT	37.01 ± 29.80	31.37 ± 31.48	29.87 ± 32.27	34.23 ± 33.87	RT-HIIT versus UC	4.55 (− 10.51, 19.61)	0.31
	AT-HIIT	31.85 ± 25.59	26.11 ± 30.74	29.00 ± 25.96	25.29 ± 21.75	AT-HIIT versus UC	− 1.82 (− 16.98, 13.34)	0.20
	UC	34.39 ± 31.79	39.88 ± 35.63	25.24 ± 28.42	22.13 ± 23.41			
Appetite loss	RT-HIIT	19.51 ± 28.89	13.73 ± 24.59	7.17 ± 17.39	3.80 ± 13.46	RT-HIIT versus UC	2.30 (− 6.10, 10.70)	− 0.03
	AT-HIIT	24.50 ± 26.70	20.00 ± 26.89	4.32 ± 13.02	5.69 ± 18.89	AT-HIIT versus UC	1.14 (− 7.35, 9.63)	− 0.16
	UC	14.81 ± 22.94	19.05 ± 27.60	6.60 ± 17.78	0.00 ± 0.00			
Constipation	RT-HIIT	21.34 ± 27.88	10.78 ± 22.63	10.76 ± 21.39	9.46 ± 17.21	RT-HIIT versus UC	0.66 (− 9.42, 10.74)	0.16
	AT-HIIT	21.44 ± 27.09	12.22 ± 21.23	9.23 ± 19.83	9.46 ± 17.21	AT-HIIT versus UC	2.21 (− 7.88, 12.30)	0.16
	UC	19.17 ± 27.23	14.88 ± 24.55	8.83 ± 19.82	2.75 ± 9.32			
Diarrhoea	RT-HIIT	14.33 ± 22.49	8.82 ± 19.63	6.89 ± 19.50	9.46 ± 20.65	RT-HIIT versus UC	3.30 (− 7.64, 14.23)	0.16
	AT-HIIT	13.15 ± 22.87	18.89 ± 28.37	4.91 ± 11.89	11.34 ± 17.89	AT-HIIT versus UC	5.21 (− 5.73, 16.16)	0.29
	UC	15.81 ± 23.85	8.33 ± 17.12	5.54 ± 14.29	7.17 ± 13.92			
Financial difficulties	RT-HIIT	21.44 ± 31.64	25.00 ± 35.21	13.78 ± 31.23	6.85 ± 21.37	RT-HIIT versus UC	− 2.22 (− 14.39, 9.95)	0.22
	AT-HIIT	16.54 ± 26.85	22.78 ± 34.98	4.94 ± 15.06	5.88 ± 17.39	AT-HIIT versus UC	1.79 (− 10.38, 13.95)	0.37
	UC	21.62 ± 33.05	19.05 ± 33.55	7.79 ± 22.20	0.00 ± 0.00			

*SD* Standard deviation, *CI* Confidence interval, *ES* Effect size, *EORTC* European organisation for research and cancer treatment Quality of life questionnaire, *RT-HIIT* Resistance training and high-intensity interval exercise group, *AT-HIIT* Moderate-intensity and high-intensity interval training group, *UC* Usual care, *ES* Effect size.

*p < 0.05.

#### Cardiorespiratory fitness, muscle strength, pain sensitivity, and body mass

No statistically significant differences were found between either exercise group and UC for cardiorespiratory fitness. However, clinically significant differences favouring the AT-HIIT group were found for both estimated VO2 peak (L⋅min^-1^): 0.18 (95% CI − 0.12, 0.47, **ES = 0.55**) and estimated VO2 peak (ml⋅kg^-1^⋅min^-1^): 1.62 (95% CI − 3.57, 6.82, **ES = 0.54**) (Table 8). Similarly, although there were no statistically significant differences between RT-HIIT and UC for lower limb muscle strength, clinically meaningful differences were seen favouring RT-HIIT: MD = 10.28, (95% CI − 2.83, 23.40, **ES = 0.52**). No statistically significant differences were observed for hand grip strength or body mass (Table 8). While no statistically significant differences were found for pain sensitivity assessed with PPT at the trapezius between the groups, there were statistically significant and clinically meaningful differences between RT-HIIT and UC favouring RT-HIIT for PPT at the gluteus: MD = 79.00 (**95% CI 10.17, 147.83, ES = 0.55**). No such differences were found for AT-HIIT vs. UC in gluteal PPT.

**Table 8:** Cardiorespiratory fitness, muscle strength, pain pressure threshold, physical activity, sedentary behaviour, and body mass 5 years post baseline.

			Baseline Mean ± SD		16 weeks Mean ± SD		2 years Mean ± SD	5 years Mean ± SD		Baseline to 5 years Between-group differences Mean difference (95% CI)	ES
Estimated VO2peak (L⋅ min^-1^)		RT-HIIT	2.25 ± 0.50		2.18 ± 0.57		2.46 ± 0.68	2.24 ± 0.58	RT-HIIT versus UC	0.15 (− 0.15, 0.44)	0.20
		AT-HIIT	2.10 ± 0.47		2.08 ± 0.49		2.20 ± 0.66	2.26 ± 0.66	AT-HIIT versus UC	0.18 (− 0.12, 0.47)	0.55
		UC	2.19 ± 0.53		1.93 ± 0.53		2.30 ± 0.53	2.08 ± 0.63			
Estimated VO2peak (ml⋅kg^-1^⋅min^-1^)		RT-HIIT	33.45 ± 7.91		31.70 ± 8.26		35.36 ± 10.14	31.82 ± 9.20	RT-HIIT versus UC	3.43 (− 1.75, 8.61)	0.06
		AT-HIIT	31.30 ± 6.65		31.36 ± 6.27		33.72 ± 10.56	32.92 ± 9.70	AT-HIIT versus UC	1.62 (− 3.57, 6.82)	0.54
		UC	32.40 ± 7.79		27.55 ± 6.64		33.24 ± 9.87	30.26 ± 7.89			
Isometric mid-thigh pull (kg)		RT-HIIT	87.23 ± 29.55		100.24 ± 34.31		105.24 ± 40.11	110.34 ± 43.01	RT-HIIT versus UC	10.28 (− 2.83, 23.40)	0.52
		AT-HIIT	78.35 ± 25.11		88.26 ± 23.02		92.76 ± 29.25	93.73 ± 31.83	AT-HIIT versus UC	− 4.31 (− 18.05, 9.42)	0.27
		UC	89.32 ± 25.27		85.81 ± 25.96		92.90 ± 26.01	98.00 ± 28.81			
Handgrip surgery side (kg)		RT-HIIT	28.40 ± 5.04		29.44 ± 5.27		29.21 ± 6.14	29.02 ± 6.62	RT-HIIT versus UC	0.37 (− 1.58, 2.33)	0.11
		AT-HIIT	28.44 ± 4.96		28.08 ± 5.29		28.20 ± 5.30	27.69 ± 5.42	AT-HIIT versus UC	− 0.06 (− 2.02, 1.89)	− 0.14
		UC	29.00 ± 6.16		27.72 ± 5.78		28.56 ± 5.18	29.02 ± 6.05			
Handgrip non-surgery side (kg)		RT-HIIT	27.71 ± 4.93		28.39 ± 5.54		28.30 ± 5.97	27.88 ± 6.34	RT-HIIT versus UC	0.34 (− 1.83, 2.51)	0.13
		AT-HIIT	27.87 ± 5.44		27.41 ± 5.48		27.61 ± 6.06	26.84 ± 6.09	AT-HIIT versus UC	0.57 (− 1.60, 2.73)	− 0.08
		UC	28.47 ± 6.50		27.18 ± 6.33		27.93 ± 5.56	27.90 ± 5.82			
Pressure pain threshold trapezius		RT-HIIT	419.19 ± 142.46		448.40 ± 149.84		401.26 ± 170.48	272.39 ± 107.65	RT-HIIT versus UC	51.07 (− 12.01, 114.15)	0.24
		AT-HIIT	402.29 ± 154.23		388.00 ± 122.79		356.71 ± 152.84	251.09 ± 78.71	AT-HIIT versus UC	41.16 (− 22.47, 104.78)	0.20
		UC	401.54 ± 134.07		366.05 ± 124.57		344.50 ± 133.38	221.17 ± 107.48			
Pressure pain threshold gluteus		RT-HIIT	420.54 ± 144.60		441.00 ± 134.38		422.57 ± 206.57	302.85 ± 114.63	RT-HIIT versus UC	79.00 (10.17, 147.83)*	0.55
		AT-HIIT	422.44 ± 196.89		413.24 ± 145.84		356.30 ± 152.83	235.76 ± 70.94	AT-HIIT versus UC	3.50 (− 69.63, 76.63)	0.06
		UC	429.03 ± 142.97		372.63 ± 140.54		353.82 ± 134.40	232.49 ± 94.44			
MVPA (min/week)		RT-HIIT	79.83 ± 31.67		–		86.99 ± 33.73	77.78 ± 37.36	RT-HIIT versus UC	3.00 (− 18.12, 24.12)	− 0.01
		AT-HIIT	70.40 ± 28.88		–		78.94 ± 33.59	70.83 ± 37.54	AT-HIIT versus UC	− 0.38 (− 21.83, 21.07)	0.07
		UC	70.04 ± 36.42		–		73.81 ± 28.24	68.15 ± 30.38			
SED (min/day)		RT-HIIT	523.26 ± 112.68		–		542.26 ± 79.03	535.59 ± 80.39	RT-HIIT versus UC	− 23.16 (− 75.80, 29.49)	0.04
		AT-HIIT	543.61 ± 109.04		–		559.23 ± 81.56	556.38 ± 83.82	AT-HIIT versus UC	− 1.46 (− 55.05, 52.14)	0.04
		UC	552.83 ± 101.93		–		559.23 ± 81.56	560.92 ± 79.21			
Body mass (kg)		RT-HIIT	68.65 ± 11.34		69.47 ± 10.56		70.46 ± 10.03	69.98 ± 8.96	RT-HIIT versus UC	− 1.45 (− 3.90, 0.99)	0.01
		AT-HIIT	67.66 ± 13.00		67.34 ± 14.45		66.41 ± 10.81	67.81 ± 12.26	AT-HIIT versus UC	− 0.36 (− 2.78, 2.06)	− 0.09
		UC	68.82 ± 11.05		71.01 ± 11.66		70.94 ± 12.73	70.05 ± 10.41			

*SD* Standard deviation, *CI* Confidence interval, *ES* Effect size, *RT-HIIT* Resistance training and high-intensity interval exercise group, *AT-HIIT* Moderate-intensity and high-intensity interval training group,

*UC* Usual care, *MVPA* Objectively measured moderate- to vigorous-intensity physical activity, *SED* Objectively measured sedentary behaviour.

*p < 0.05.

#### Sedentary behaviour and physical activity

There were no statistically significant differences observed between either of the exercise groups and the usual care group in terms of minutes of sedentary behavior or moderate to vigorous PA (Table 8). Although not statistically significant, all three groups decreased their MVPA compared to the 2-year assessment point returning to their levels at baseline (Table 8).

#### Sick leave

At 5 years, as many as 97%, 86%, and 100% reported that they were not on any sick leave among RT-HIIT, AT-HIIT and UC respectively. Only 1 participant (3%) in the RT-HIIT group reported being on full-time sick leave and 3 participants (8%) in the AT-HIIT group reported being on some level of sick leave, among which 1 participant was on 25% sick leave and 2 were on 50% sick-leave.

#### Cognitive functioning

No statistically significant differences were found between the two intervention groups and the UC group for any of the cognitive tests (Table 9).

**Table 9:** Cognitive functioning 5 years post-baseline.

Amsterdam cognition Scan (ACS)		Baseline Mean ± SD		Between-group differences Mean difference (95% CI)	ES
Wordlist learning (Total numbers correct)	RT-HIIT	49.39 ± 6.97	RT-HIIT versus UC	2.11 (− 6.83, 11.04)	0.22
	AT-HIIT	47.12 ± 14.15	AT-HIIT versus UC	− 0.17 (− 8.96, 8.63)	− 0.02
	UC	47.29 ± 9.64			
Wordlist recall (Total numbers correct)	RT-HIIT	9.67 ± 3.45	RT-HIIT versus UC	1.17 (− 1.74, 4.08)	0.31
	AT-HIIT	9.22 ± 3.70	AT-HIIT versus UC	0.72 (− 2.05, 3.49)	0.19
	UC	8.50 ± 3.81			
Wordlist recognition (Total numbers correct)	RT-HIIT	29.10 ± 1.09	RT-HIIT versus UC	− 0.09 (− 0.95, 0.76)	− 0.09
	AT-HIIT	29.19 ± 1.11	AT-HIIT versus UC	− 0.00 (− 0.82, 0.81)	0
	UC	29.19 ± 0.98			
Box tapping (Total numbers correct)	RT-HIIT	8.41 ± 2.22	RT-HIIT versus UC	− 0.40 (− 1.86, 1.05)	− 0.21
	AT-HIIT	8.30 ± 1.44	AT-HIIT versus UC	− 0.52 (− 1.92, 0.88)	− 0.27
	UC	8.81 ± 1.91			
Digit sequences I (Total numbers correct)	RT-HIIT	10.65 ± 2.10	RT-HIIT versus UC	0.71 (− 1.03, 2.46)	0.30
	AT-HIIT	9.81 ± 2.27	AT-HIIT versus UC	− 0.12 (− 1.81, 1.57)	− 0.06
	UC	9.94 ± 2.35			
Digit sequences II (Total numbers correct)	RT-HIIT	8.26 ± 2.63	RT-HIIT versus UC	− 0.05 (− 2.06, 1.96)	− 0.18
	AT-HIIT	8.12 ± 2.32	AT-HIIT versus UC	− 0.20 (− 1.96, 2.06)	− 0.07
	UC	8.31 ± 2.85			
Reaction speed (Reaction time in ms)^a^	RT-HIIT	315.54 ± 57.63	RT-HIIT versus UC	− 10.92 (− 52.59, 30.76)	− 0.17
	AT-HIIT	311.36 ± 40.87	AT-HIIT versus UC	− 15.09 (− 55.11, 24.92)	− 0.24
	UC	326.46 ± 63.14			
Connect the dots I (Completion time in s) ^a^	RT-HIIT	40.57 ± 6.71	RT-HIIT versus UC	1.47 (− 4.79, 7.74)	0.17
	AT-HIIT	42.46 ± 8.07	AT-HIIT versus UC	3.37 (− 2.95, 9.68)	0.40
	UC	39.10 ± 8.49			
Connect the dots II (Completion time in s) ^a^	RT-HIIT	72.77 ± 17.60	RT-HIIT versus UC	− 1.79 (− 15.70, 12.13)	− 0.07
	AT-HIIT	67.07 ± 12.03	AT-HIIT versus UC	− 7.48 (− 21.17, 6.21)	− 0.31
	UC	74.55 ± 23.87			
Place the beads (Number of extra moves) ^a^	RT-HIIT	31.74 ± 16.95	RT-HIIT versus UC	5.11 (− 6.35, 16.58)	0.54
	AT-HIIT	27.63 ± 15.07	AT-HIIT versus UC	1.00 (− 10.11, 12.12)	0.11
	UC	26.63 ± 9.46			
Fill the grid (Completion time in s)^a^	RT-HIIT	68.16 ± 12.97	RT-HIIT versus UC	− 1.22 (− 11.93, 9.49)	− 0.10
	AT-HIIT	73.20 ± 13.77	AT-HIIT versus UC	3.82 (− 6.46, 14.09)	0.31
	UC	69.38 ± 12.36			

*SD* Standard deviation, *CI* Confidence interval, *RT-HIIT* Resistance training and high-intensity interval exercise group, *AT-HIIT* Moderate-intensity and high-intensity interval training group, *UC* Usual care. ^a^ Higher scores indicate worse performance.

## Discussion

This study examined the lasting impacts of the OptiTrain exercise program on breast cancer survivors 5 years post-intervention. The RT-HIIT group showed significant improvement in gluteal PPT compared to the control group. Clinically meaningful improvements were also seen for lower limb strength for the RT-HIIT and cardiorespiratory fitness for the AT-HIIT compared to UC group, albeit without statistical significance. Clinically significant increase in cognitive CRF was seen among the AT-HIIT group vs the UC.

Evidence shows that breast cancer survivors experience several long-term functional changes, such as reduced strength, aerobic capacity, and mobility, compared to their peers in the same age group^27^. Moreover, despite generally comparable QoL to the general population 5 years post-diagnosis, many disease-free breast cancer survivors still faced adverse effects, necessitating ongoing screening and support for fatigue, sleep, cognitive issues, pain, menopausal/sexual symptoms, physical performance, and weight problems during and after therapy^28^.

However, in the present study a clinically relevant difference for cognitive CRF sub-scale favoring the UC group was observed in the AT-HIIT group compared to UC at 5 years but lacked statistical significance. These results are in contrast with the previous findings^14,17^ of the OptiTrain trial where cognitive CRF was significantly lower among the intervention groups (AT-HIIT at the 1-year and RT-HIIT at the 2 year-follow-up mark). In fact, the fatigue scores for the total CRF and the four fatigue dimensions in the control group were lower than their baseline scores, that possibly imply a generally low level of fatigue. Moreover, none of the 3 allocation groups had an average fatigue severity score above 3, implying little remaining fatigue. Given that fatigue is a persistent side effect of cancer treatment, these are promising results for sustainable long-term effects.

No differences in cognitive functioning and subjective cognitive functioning were found between the exercise groups and the UC group at 5 years. These findings are in line with other studies that also found no long-term beneficial effects of PA on objective cognitive functioning^29,30^. Because cognitive functioning was assessed only at 5 years, we cannot correct for possible baseline differences between groups nor draw any conclusion about change in cognitive functioning over time. An ongoing follow-up study assessing cognitive functioning at baseline prior to neoadjuvant chemotherapy and 1 year later, will allow further investigation on whether physical exercise during chemotherapy for breast cancer could have any potential protective effect on cognition^31^.

At 5 years, the AT-HIIT group demonstrated clinically relevant differences in cardiorespiratory fitness compared to the control group but lacked statistical significance and may be attributed to lack of statistical power with only 40% of those who were randomised from the OptiTrain participants remained at 5 years. These results are in line with previous findings of the OptiTrain trial^15–17^ and other studies that show that aerobic training results in sustained cardiorespiratory fitness^32^. Similarly, the RT-HIIT group still showed clinically meaningful differences for leg muscle strength again without statistical significance potentially owing to the slightly diminished statistical power. Muscle strength is vital for patients, as it is linked to cancer-related fatigue and overall survival in older breast cancer survivors^33,34^. Furthermore, it's more critical than muscle mass in conditions like sarcopenia, which heightens the risk of falls, reduces independence, lowers quality of life, and shortens lifespan^35^. Though additional research is required to confirm our initial results, suggesting that engaging in specific exercise during chemotherapy could be a promising way to improve cardiorespiratory fitness and lower body muscle strength for up to five years, these findings warrant attention. We speculate that these may partly result from the lasting habit formation and knowledge gained during the supervised exercise intervention.

Participants in the RT-HIIT group reported favourable statistically and clinically significant differences in PPT gluteus than the UC group. These results are particularly promising as a higher percentage of participants at the 5-year assessment had received taxane-based therapy as their treatment regimen compared to the previous assessment points of the OptiTrain study (Table 3). Taxane-based therapy is now crucial in treating early-stage and advanced cancers, including breast carcinoma, and has led to increased survival rates^36^. Over the past decade, it has become widely used in breast cancer management. However, it comes with side effects like peripheral neuropathy, myelosuppression, joint and muscle pain, and skin reactions, which can negatively affect participants' quality of life^37–40^. Particularly, peripheral neuropathy can be severe in patients undergoing taxane therapy which may increase numbness, leading to decreased PPT^38,40^. This is promising because our prior findings at 16 weeks showed that the RT-HIIT group on taxane treatment did not become more sensitive to pain over time^15^. It's particularly encouraging that even 5 years after completing the exercise program, they continue to experience improved pain sensitivity in the gluteus, a significant muscle group for the lower extremities. Our study is the first to show that participating in specific exercise during chemotherapy for breast cancer shows promise as an effective method to reduce pain sensitivity in the gluteus even up to 5 years after the intervention. Despite the lack of statistical link between changes in gluteus PPT and lower-limb muscle strength (data not shown), evidence suggests a correlation between muscle strength and PPT in both healthy and diseased populations^41,42^. The RT-HIIT group's continued resistance to increased pain sensitivity at the gluteus, but not the trapezius, may be due to their sustained higher lower-limb muscle strength, which remained significantly greater even five years post-intervention. We speculate that this difference may be partly due to the long-term benefits of exercise habit formation and knowledge gained from the supervised resistance exercise program.

At the 5-year point, no significant differences in PAL were observed among the three groups. Moreover, all three groups reduced their MVPA levels compared to the assessment at 2 years, returning to baseline levels. In this study only 2 participants (3%) met the current PA recommendations of 150 minutes of MVPA per week. This suggests a potential decline in the long-term exercise adherence effects and habits developed during the supervised resistance exercise program, likely due to the absence of extended motivational support beyond the 2-year assessments to foster exercise autonomy among participants and the attendance rate at the motivational seminars up to the 2- year follow-up was already low. At the 5-year mark, most participants in all three groups self-reported a moderate activity level (57% in RT-HIIT, 58% in AT-HIIT, and 58% in UC, data not shown). Our findings contrast with those of the PACT study and the study by Mutrie et al, where the exercise intervention groups showed higher PAL four- and five-years post-intervention, respectively^6,7^. It is noteworthy that the methods for measuring PA differed between the studies (questionnaires vs accelerometers), making direct comparisons challenging.

Notable strengths of the present study include the long follow-up period with prospective data collection and the use of validated questionnaires. While prospective data collection minimizes selection bias, some potential bias might remain due to a reduced sample size of 95 participants at the 5-year assessment, mostly due to participant attrition. In experimental studies, where costs often constrain sample sizes, having 95 individuals for long-term follow-up can still be considered an acceptable attrition rate. Another strength is the use of accelerometers for objective, long-term PA and sedentary behavior measurement in a randomized controlled trial lasting up to 5 years that overcomes issues associated with self-reported questionnaires^43^. However, it's essential to recognize that accelerometers may not fully capture certain activities like resistance exercise, cycling, or water sports, particularly when worn on the hip. Additionally, a limitation of the study is the lack of specific details about the types of physical activities participants engaged in during the follow-up, potentially leading to an underestimate of overall PA levels, particularly during assessments.

## Conclusions

Notably in our study, the RT-HIIT group exhibited substantial enhancements in gluteal PPT compared to UC. Additionally, clinically significant improvements were observed in lower limb strength for RT-HIIT and cardiorespiratory fitness for the AT-HIIT compared to UC, but an increase in cognitive CRF although statistical significance was not attained. However, it also highlights a concerning decline in physical activity levels over time. These findings provide new perspectives on the long-term benefits of incorporating an exercise program during chemotherapy for women in the early- curative setting with breast cancer. Nonetheless, the study also emphasizes the importance of developing enhanced strategies to assist survivors in maintaining their PAL and offering continuous motivational support and PA prescriptions support throughout their survivorship journey.

## Data Availability

The datasets used and analysed during the current study are available from the corresponding author on reasonable request.
